# Deep Learning-Based Banknote Fitness Classification Using the Reflection Images by a Visible-Light One-Dimensional Line Image Sensor

**DOI:** 10.3390/s18020472

**Published:** 2018-02-06

**Authors:** Tuyen Danh Pham, Dat Tien Nguyen, Wan Kim, Sung Ho Park, Kang Ryoung Park

**Affiliations:** Division of Electronics and Electrical Engineering, Dongguk University, 30 Pildong-ro 1-gil, Jung-gu, Seoul 100-715, Korea; phamdanhtuyen@dongguk.edu (T.D.P); nguyentiendat@dongguk.edu (D.T.N.); daiz0128@naver.com (W.K.); 01072669850@naver.com (S.H.P.)

**Keywords:** fitness classification, deep learning, reflection images of banknote, visible-light one-dimensional line image sensor, convolutional neural network

## Abstract

In automatic paper currency sorting, fitness classification is a technique that assesses the quality of banknotes to determine whether a banknote is suitable for recirculation or should be replaced. Studies on using visible-light reflection images of banknotes for evaluating their usability have been reported. However, most of them were conducted under the assumption that the denomination and input direction of the banknote are predetermined. In other words, a pre-classification of the type of input banknote is required. To address this problem, we proposed a deep learning-based fitness-classification method that recognizes the fitness level of a banknote regardless of the denomination and input direction of the banknote to the system, using the reflection images of banknotes by visible-light one-dimensional line image sensor and a convolutional neural network (CNN). Experimental results on the banknote image databases of the Korean won (KRW) and the Indian rupee (INR) with three fitness levels, and the Unites States dollar (USD) with two fitness levels, showed that our method gives better classification accuracy than other methods.

## 1. Introduction

The functionalities of sorting and classifying paper currency in automated transaction facilities, such as automated teller machines (ATMs) or counting machines consist of the recognition of banknote types, denominations, counterfeit detection, serial recognition, and fitness classification [1]. The fitness classification of banknotes is concerned with the evaluation of the banknotes’ physical conditions, such as staining, tearing, or bleaching. This task helps not only to determine whether a banknote is suitable for recirculation or should be replaced by a new one, but also to enhance the processing speed and sorting accuracy of the counting system. 

Fitness of banknotes is normally classified based on the banknotes’ optical characteristics captures by imaging sensors. In general, the presentations of banknotes are different among types of banknotes as well as between front and back sides of the banknote itself. As a result, fitness classification of banknote proposed in most previous studies was performed under the assumption that the input banknote’s type, denomination, and input direction are known [1]. In the next Section, we provide detailed explanations of the related work concerning banknote fitness classification.

## 2. Related Works

Studies on banknote fitness classification with regard to various paper currencies have been reported. According to the research by the Dutch central bank, De Nederlandsche Bank (DNB), based on the evaluation using color imaging, soiling was the predominant reason that degrades the quality of a banknote, and the mechanical defects appeared after the banknote was stained [2,3,4]. Therefore, several previous studies use the soiling level as the criterion for judging the fitness for further circulation of a banknote [5]. Based on the method of using banknote images captured by single or multiple sensors, these approaches can be divided into two categories: the methods using the whole banknote image and those that use certain regions of interest (ROIs) on the banknote image for the classification of banknote fitness. In the method proposed by Sun and Li [6], they considered that the banknotes with different levels of old and new have different gray histograms. Therefore, they used the characteristics of the banknote images’ histogram as the features, dynamic time warp (DTW) for histogram alignment, and support vector machine (SVM) for classifying the banknotes’ age. Histogram features were also used in the research of He et al. [7], in which they used a neural network (NN) as the classifier. A NN was also used in the Euro banknote recognition system proposed by Aoba et al. [8]. In this study, the whole banknote images captured by visible and infrared (IR) sensors were converted to multiresolutional input values and subsequently fed to the classification part using a three-layered perceptron and the validation part uses the radial basis function (RBF) networks [8]. In this system, the new and dirty Euro banknotes are classified in the RBF network-based validation part. Recently, Lee et al. [9] proposed a soiled banknote determination based on morphological operations and Otsu’s thresholding on contact image sensor (CIS) images of banknotes.

In ROI-based approaches, certain areas on the banknote images where the degradation can be frequently detected or visualized are selected for evaluating the fitness of the banknote. In the studies of Geusebroek et al. [3] and Balke et al. [10], from overlapping rectangular regions on the color images of Euro banknotes, the mean and standard deviation of the channels’ intensity values were calculated and selected as the features for assessing the soiling values of banknotes using the AdaBoost algorithm [3,10]. Mean and standard deviation values of the wavelet-transformed ROIs were also the classification features in the method proposed by Pham el al. [11]. In this study, these features were extracted from the little textures containing areas on the banknote images using discrete wavelet transform (DWT) and selected based on a correlation with the densitometer data and subsequently used for fitness classification by the SVM [11]. The regions with the least amount of textures are also selected for feature extraction in the study proposed by Kwon et al. [12], in which they used both the features extracted from visible-light reflection (VR) and near-infrared light transmission (NIRT) images of the banknotes, and the fuzzy-based classifier for the fitness classification system.

The methods that are based on certain regions on the banknotes for evaluating the fitness of banknotes have advantages of reduced input data size and processing time. However, the selection of ROIs in the previous fitness classification studies is mostly manual, and the degradation and damage of banknote can occur on the unselected areas. The global-feature-based banknote images could help to solve this problem, but since the input features are mostly based on the brightness characteristic of the banknote images, it is much affected by illumination change, wavelength of sensors, and variation in patterns of different banknote types. Moreover, in fitness classifications, most studies assumed that the input banknote’s type, denomination, and input direction are known [1].

To overcome these shortcomings, we considered a method for classification of banknote fitness based on the convolutional neural network (CNN). This NN structure was first introduced by LeCun et al. in their studies about handwritten character recognition [13,14], and have recently been emerging and attracting research interest [15], especially for the image classification of the ImageNet large-scale visual recognition challenge (ILSVRC) contest [16,17,18,19]. However, little research has been conducted on the automatic sorting of banknotes using CNNs. Ke et al. proposed a banknote image defect detection method using a CNN [20]; however, this study had only focused on the recognition of ink dots in banknote image defects, and did not specify the type of experimental banknote image dataset or judge the fitness for recirculation of the examined banknotes. Another recent CNN-based method proposed by Pham et al. [21] aiming to classify banknote type, denomination, and input direction showed good performance even with the mixed dataset from multiple national currencies. On the evaluation of a state-of-the-art method, we proposed a deep learning-based banknote fitness-classification method using a CNN on the gray-scale banknote images captured by visible-light one-dimensional line image sensor. Our proposed system is designed to classify the fitness of banknote into two or three levels including: (i) fit and unfit, and (ii) fit, normal and unfit for recirculation, depending on the banknote’s country of origin, and regardless of the denomination and input direction of the banknote. Compared to previous studies, our proposed method is novel in the following aspects:

(1) This is the first CNN-based approach for banknote fitness classification. We performed training and testing of a CNN on banknote image databases of three national currencies that consist of 12 denominations, by which the performance of our proposed method is confirmed to be robust to a variety of banknote types.
(2) Our study carried out fitness determination on the United States dollar (USD), the Korean won (KRW), and the Indian rupee (INR), in which three levels of fitness of banknote, namely fit, normal, and unfit cases for recirculation, are considered with the KRW and INR, whereas two levels of fit and unfit cases are considered with the USD.
(3) Our fitness recognition system can classify the fitness of banknote regardless of the denomination and direction of the input banknote. As a result, the pre-classification of banknote image in the denomination and input direction is not required, and there is only one trained fitness-classification model for each national currency.
(4) We made our trained CNN model with databases publicly available by other researchers for the fair comparisons with our method and databases.

Table 1 gives a comparison between our research and previous studies. The details of the proposed banknote fitness-classification method are presented in Section 3. Experimental results and conclusions are given in Section 4 and Section 5 of this paper, respectively.

## 3. Proposed Method 

### 3.1. Overview of the Proposed Method

**Figure 1:**
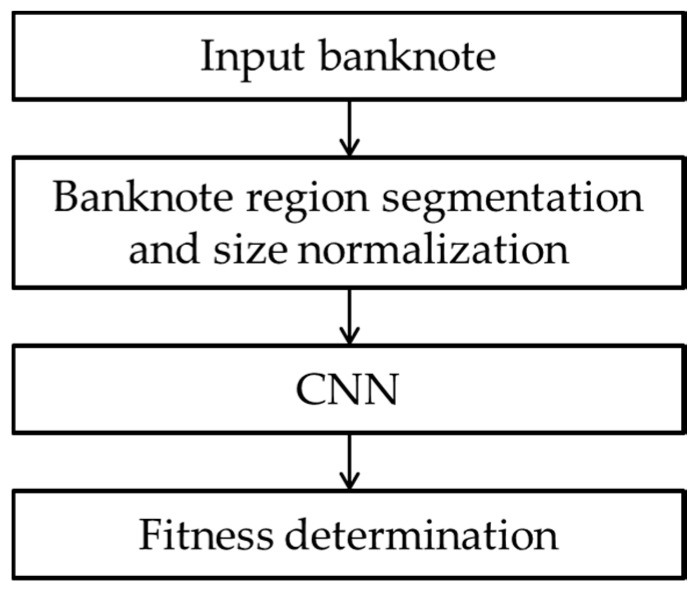
Overall flowchart of the proposed method.

The overall flowchart of the proposed method is shown in Figure 1. The input banknote image is captured and pre-processed. In this pre-processing step, the banknote region in the captured image by visible-light one-dimensional line image sensor is segmented from the background and resized to achieve the same size of 115 × 51 pixels, because the size of the input image to the CNN should be the same. The size-normalized image of the banknote is fed into the pre-trained CNN, and the level of fitness is determined at the output of the network.

### 3.2. Acquisition and Pre-Processing of Banknote Image

For banknote image acquisition in this study, we used a commercial banknote counting machine with a visible-light one-dimensional line image sensor that has a resolution of 1584 pixels [12,22]. A line sensor was used instead of the conventional two-dimensional (area) image sensors because of the size limitation and the cost of the counting machine. When a banknote is input to the system, it will be passed through the rollers inside the machine and illuminated by visible-light light-emitting diode (LED), and the line sensor is triggered successively at a high speed to capture the line images of the input banknote. The number of trigger times when the input banknote is a KRW or INR is 464, meanwhile that in the case of the USD it is 350. By concatenating the captured line images, the resulting acquired banknote image has a resolution of 1584 × 464 pixels or 1584 × 350 pixels in the case of the KRW-INR banknote or the USD banknote, respectively.

**Figure 2:**
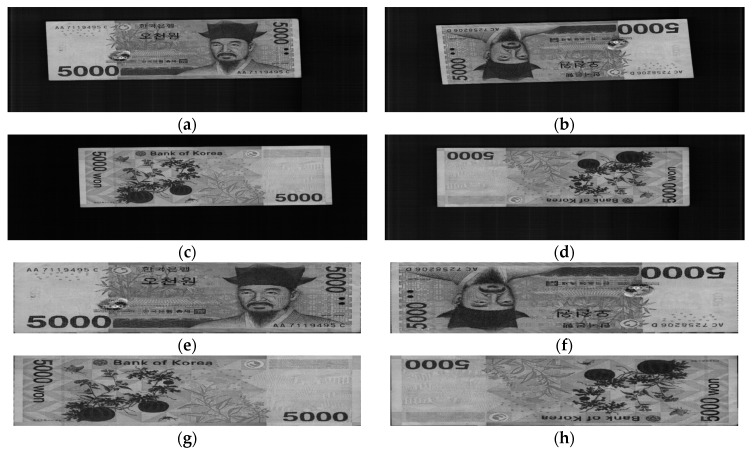
Example of input banknote images in four directions: Original captured banknote image in (**a**) A direction; (**b**) B direction; (**c**) C direction; (**d**) D direction; (**e**–**h**) Corresponding banknote region segmented from the images in (**a**–**d**), respectively.

Four input directions of the banknotes when being inserted into the counting machine are labeled as A, B, C, and D, which are the front side in the forward direction, front side in the backward direction, back side in the forward direction, and back side in the backward direction, respectively. Examples of banknote images in the A to D directions in the case of the KRW are shown in Figure 2. The original banknote image captured by the counting machine includes both the banknote region and surrounding background. By using the corner detection algorithm built into the counting machine, we segment the banknote region from the background to address the area that contains meaningful information of the banknote image, as well as fix the displacement and rotation of the input banknote, as shown in Figure 2. The detail explanations of the corner detection algorithm are as follows. Within the fixed ROI of the captured banknote image of Figure 2a–d, the upper boundary of banknote is detected by scanning a one-dimensional mask for edge detection based on the 1st order derivative [23] from upper to lower position per each horizontal position of the ROI. From this, the candidate points of upper boundary are detected, and accurate boundary line is determined by line fitting algorithm [23] with these points. Same procedure is iterated for detecting lower, left, and right boundaries of banknote. Left boundary is detected by scanning the same mask from left to right position per each vertical position of ROI for detecting left boundary whereas right one is detected by scanning same mask from right to left position per each vertical position of ROI for detecting right boundary. Then, four boundary lines are located, and the four intersected points by these lines are determined as the corner points of banknote. The segmented banknote images are then resized equally to achieve the same size of 115 × 51 pixels to be inputted to the CNN in the next step.

### 3.3. The CNN Architecture

**Figure 3:**
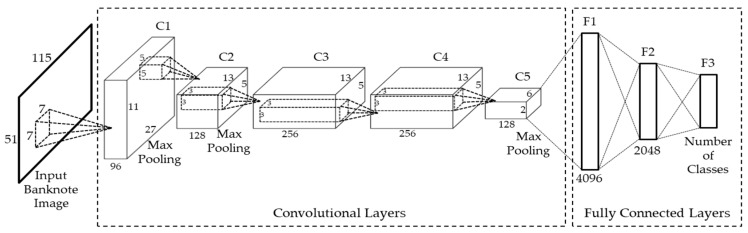
Convolutional neural network (CNN) architecture used in our proposed method.

The CNN architecture used in our proposed method is shown in Figure 3 and Table 2. This network structure consists of five convolutional layers, denoted as C1 to C5, followed by three fully connected layers, denoted as F1 to F3, which are similar to those in the AlexNet architecture [16,21]. For faster training time with gradient descent, rectified linear unit (ReLU) layers are presented at all of the convolutional layers and fully connected layers of the network [16]. Using the ReLU activation function, whose formula is shown in Equation (1), instead of the standard non-linear function of the sigmoid or hyperbolic tangent, as shown in (2) and (3), respectively, can help to avoid the gradient-vanishing effect [24]:

(1) f(x)=max(x,0)

(2) $$f(x)=\frac{1}{1+e^{-x}}$$

(3) f(x)=tanh(x)

Local response normalization is considered at the first two layers of Conv1 and Conv2 with cross-channel normalization (CCN) layers [16,21], whose equation is presented follows:

(4) $${a^{\prime }}_{x,y}^{i}=\frac{a_{x,y}^{i}}{{\left(k+\alpha \sum_{j=\max(0,i-\frac{n}{2})}^{\min(N-1,i+\frac{n}{2})}{\left(a_{x,y}^{j}\right)}^{2}\right)}^{\beta }}$$

where $a_{x,y}^{i}$ is the neuron activity computed by applying the kernel *i*th at position (*x*, *y*). With the normalization executed for the adjacent *n* kernel maps at the same spatial position, the obtained normalized activity value is $a_{x,y}^{\prime i}$. In Equation (4), *N* is the total number of kernels in the layer. We choose a window channel size *n* of 5; *k*, *α*, and *β* are hyper-parameters and are set to 1, 0.0001, and 0.75, respectively. In Equation (4), the term of summation of ${\left(a_{x,y}^{j}\right)}^{2}$ multiplied by *α* can be zero in case that all the ${\left(a_{x,y}^{j}\right)}^{2}$ are zero. Therefore, the off-set value of *k* is used in order to make the denominator of Equation (4) non-zero. *α* is the kind of control parameter. For example, if the term of summation of ${\left(a_{x,y}^{j}\right)}^{2}$ multiplied by *α* is much larger than *k*, $a_{x,y}^{\prime i}$ of Equation (4) approximates $a_{x,y}^{i}$/(the term of summation of ${\left(a_{x,y}^{j}\right)}^{2}$ multiplied by *α*) by ignoring *k*. On the contrary, if the term of summation of ${\left(a_{x,y}^{j}\right)}^{2}$ multiplied by *α* is much smaller than *k*, $a_{x,y}^{\prime i}$ of Equation (4) approximates $a_{x,y}^{i}$/*k* by ignoring the term of summation of ${\left(a_{x,y}^{j}\right)}^{2}$ multiplied by *α*. *β* is also the kind of control parameter. With larger *β*, the $a_{x,y}^{\prime i}$ becomes smaller whereas the $a_{x,y}^{\prime i}$ becomes larger with smaller *β*. The *k*, *α*, and *β* are also called as hyper-parameters based on previous researches [16]. The optimal values (1, 0.0001, and 0.75) of these parameters were experimentally determined with training data.

Following each CNN layer in the first and second convolutional layer is the max pooling layer. The max pooling is also adopted in the last convolutional layer (C5) before connecting to the fully connected layer part of the network structure. The gray-scale banknote images in our proposed method are resized equally to 115 × 51 pixels using linear interpolation before being fed into the CNN. Through each layer of the network structure, feature map size changes are as shown in Table 2 according to the following equations [21,25]:

(5) $$w_{i}=\frac{w_{i-1}-w_{F}+2p}{s}+1$$

(6) $$h_{i}=\frac{h_{i-1}-h_{F}+2p}{s}+1$$

(7) $$c_{i}=\{\begin{array}{cc} c_{i-1} & \;\mathrm{for}\;i\mathrm{th}\;\mathrm{pooling}\;\mathrm{layer}\\ k & \;\mathrm{for}\;i\mathrm{th}\;\mathrm{convolutional}\;\mathrm{layer} \end{array}$$

where *w_i_*, *h_i_*, and *c_i_*, denoting the width, height, and number of channels, respectively, are the sizes of the feature map in the *i*th convolutional layer in pixels; those of its preceding (*i* − 1)th layer are denoted as *w_i_*_−1_, *h_i_*_−1_, and *c_i_*_−1_; the *i*th layer has *k* filters with the number of weights per filter is (*w_F_* × *h_F_* × *c_i_*), the filtering stride is *s* pixels, and the zero-padding amount is *p* pixels. The resulting banknote feature map after five convolutional layers has the size of 6 × 2 × 128 = 1536, as shown in Table 2, and these features are fed into the fully connected layers of the network.

To prevent the overfitting problem, we inserted a dropout layer between the 2nd and 3rd fully connected layers, as shown in Table 2. This is the regularization method that randomly disconnects the neuron unit from the network during training [16,26]. *p* is the probability of maintaining the connections. For example, if there are 100 connections of the neuron unit from the network, 35 connections are randomly disconnected with the *p* of 0.65 (the connections of 65% are maintained). In this research, we chose *p* equal to 0.65. The optimal value (0.65) of *p* was experimentally determined with training data. In order to do so, the input vector **y** to the network node is element-wise multiplied with a vector **r** consisting of the independent Bernoulli random variables, each of which can be 0 or 1 with the probability *p* [26]. Therefore, **r** ~ Bernoulli(*p*) [26]. For example, if **y** of Equation (8) has the 100 components of (*y*_1_, *y*_2_, …, *y*_100_), the **r** has the 100 components of (*r*_1_, *r*_2_, …, *r*_100_), also, for the element-wise multiplication of **y** and **r** (“•” of Equation (8)). If the probability *p* is 0.65, 65 components of (*r*_1_, *r*_2_, …, *r*_100_) are 1 and the remained 35 ones are 0. *z* of Equation (8) stands for the output of feed-forward operation of the neuron unit with dropout, activation function *f*(·), weights of **w**, and bias *b*:

(8) z=f(w(y•r)+b)

As mentioned above, banknote features are completely extracted at the output of the final 5th convolutional layer. The fully connected layers that follow can be considered as the classifier part of the CNN structure. The number of network nodes in the three fully connected layers (F1 to F3) in our study is shown in Table 2. In this research, we classified banknote fitness to three levels in the case of the KRW and INR, and two levels for the USD banknotes. As a result, the number of nodes in the last fully connected layer may vary according to the national currency selected.

At the output stage of the CNN structure, we apply a normalized exponential function (softmax function) [27] that helps to transform the real values at the outputs of the neuron units in F3 to the values in the range of (0, 1). These resulting values of the softmax function can be considered as the probability that the input banknote belongs to the fitness classes corresponding to the network outputs. The softmax layer can also help to highlight the largest values and suppress the smaller values among the set [21]. The formula of the softmax function applied on the node output values denoted as *z_i_* is shown in the following Equation (9):

(9) $$p_{i}=\frac{e^{z_{i}}}{\sum_{i=1}^{N}e^{z_{i}}}$$

Among *N* fitness levels, the one corresponding to the maximum value of *p_i_* (*i* = 1, …, *N*) is considered as the fitness level of the input banknote image. In this research, the training process for the filter parameters of convolutional layers and the network weights of fully connected layers are conducted separately for each national currency of KRW, INR, and USD, in combination of all the denominations and input directions of the banknote images. By conducting this training on the CNN model, our proposed fitness-classification method does not require the pre-classification of the denomination type and direction of the banknote. The completely trained CNN models are stored in the memory for use in the testing experiments.

## 4. Experimental Results

**Figure 4:**
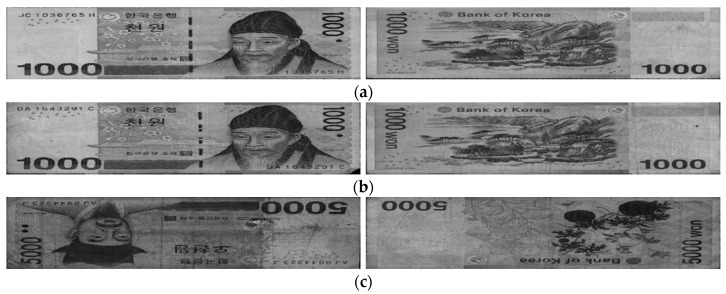
Example of banknote images in the KRW database with fitness levels of (**a**) Fit, (**b**) Normal, and (**c**) Unfit.

**Figure 5:**
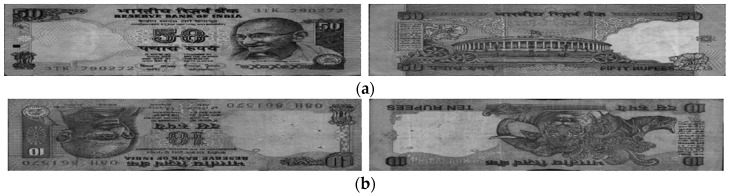

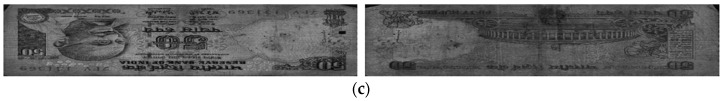
Example of banknote images in the INR database with fitness levels of (**a**) Fit, (**b**) Normal, and (**c**) Unfit.

**Figure 6:**
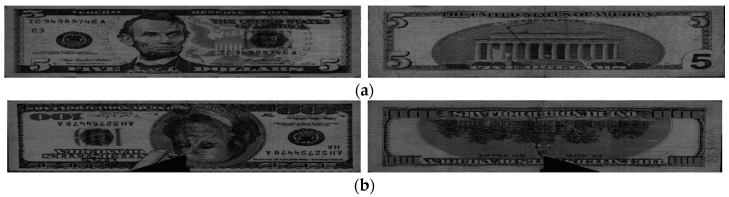
Example of banknote images in the USD database with fitness levels of (**a**) Fit and (**b**) Unfit.

We used banknote fitness databases from three national currencies, which are the KRW, INR, and USD, for the experiments using our proposed method. The KRW banknote image database is composed of banknotes in two denominations, 1000 and 5000 wons. The denominations of banknotes in the INR database are 10, 20, 50,100, 500, and 1000 rupees. Those for the case of the USD are 5, 10, 50, and 100 dollars. Three levels of fitness, which are fit, normal, and unfit for recirculation, are assigned for the banknotes of each denomination in the cases of the KRW and INR, and two levels including fit and unfit are defined for the USD banknotes in the experimental dataset. Examples of banknotes assigned to each fitness level are shown in Figure 4, Figure 5 and Figure 6. 

The number of banknotes in each fitness level of three national currency databases is given in Table 3. We made our trained CNN model with databases publicly available by other researchers through [28] for the fair comparisons with our method and databases.

We conducted the experiments using the two-fold cross-validation method. Therefore, the dataset of banknote images from each national currency was randomly divided into two parts. In the first trial, one of the two parts was used for training, and the other was used for testing. The process was repeated with these parts of the dataset swapped in the second trial. With the obtained results from two trials, we calculated the overall performance by averaging two accuracies.

In this research, we trained the network models separately for each national currency dataset without pre-classifying the denomination and input direction of the banknote images in the dataset. In each dataset, we performed data augmentation for expanding the number or image for training. This process helps to generalize the training data and reduce overfitting [21]. For data augmentation, we randomly cropped the boundaries of the original image in the dataset in the range of 1 to 7 pixels. The number of images in the datasets of the KRW and INR were increased by multiplication factors of 3 and 6 times, respectively. In the case of the USD, the numbers of fit and unfit banknote images were multiplied by 21 and 71 times. Consequently, the total number of images for training in each national currency dataset was approximately 100,000 images. We also listed the number of images in each dataset and each class after augmentation in Table 3.

**Figure 7:**
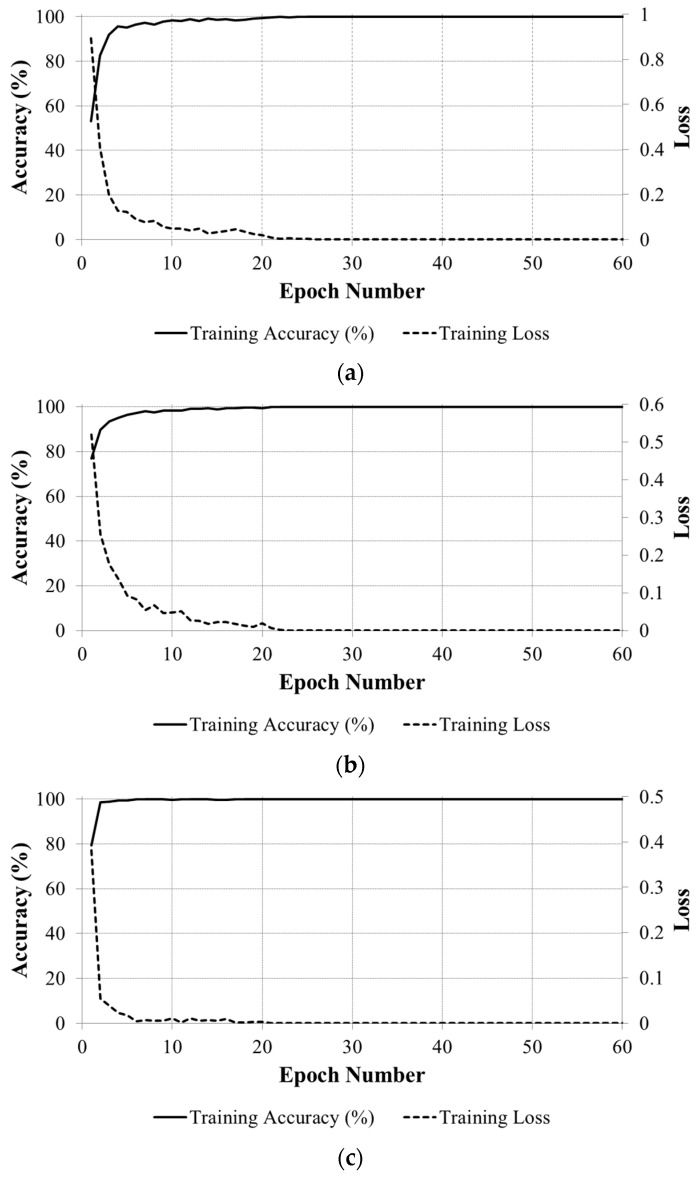
Convergence graphs with average accuracies and losses according to the epoch number on two subsets of training data in two-fold cross-validation on each national currency dataset: (**a**) KRW; (**b**) INR; and (**c**) USD.

In the first experiments of the CNN training, we trained three network models for fitness classification in each of the national currency dataset, and repeated it twice for two-fold cross-validation. Training and testing experiments were performed using the MATLAB implementation of the CNN [29] on a desktop computer equipped with an Intel^®^ Core™ i7-3770K CPU @ 3.50 GHz [30], 16-GB memory, and an NVIDIA GeForce GTX 1070 graphics card with 1920 CUDA cores, and 8-GB GDDR5 memory [31]. The training method is stochastic gradient descent (SGD), also known as sequential gradient descent, in which the network parameters are updated based on the batch of data points at a time [27]. The CNN training parameters were set as follows: the number of iterations for training is 60 epochs, with the initial learning rate of 0.01 and reduced by 10% at every 20 epochs. The convergence graphs of the average batch loss and accuracy according to the epoch number of the training process on the two subsets of training data in the two-fold cross-validation are shown in Figure 7 for each country’s banknote dataset. Figure 7 shows that the accuracy values increased to 100% and the loss curves approach zero with the increment of epoch number in all cases.

**Figure 8:**
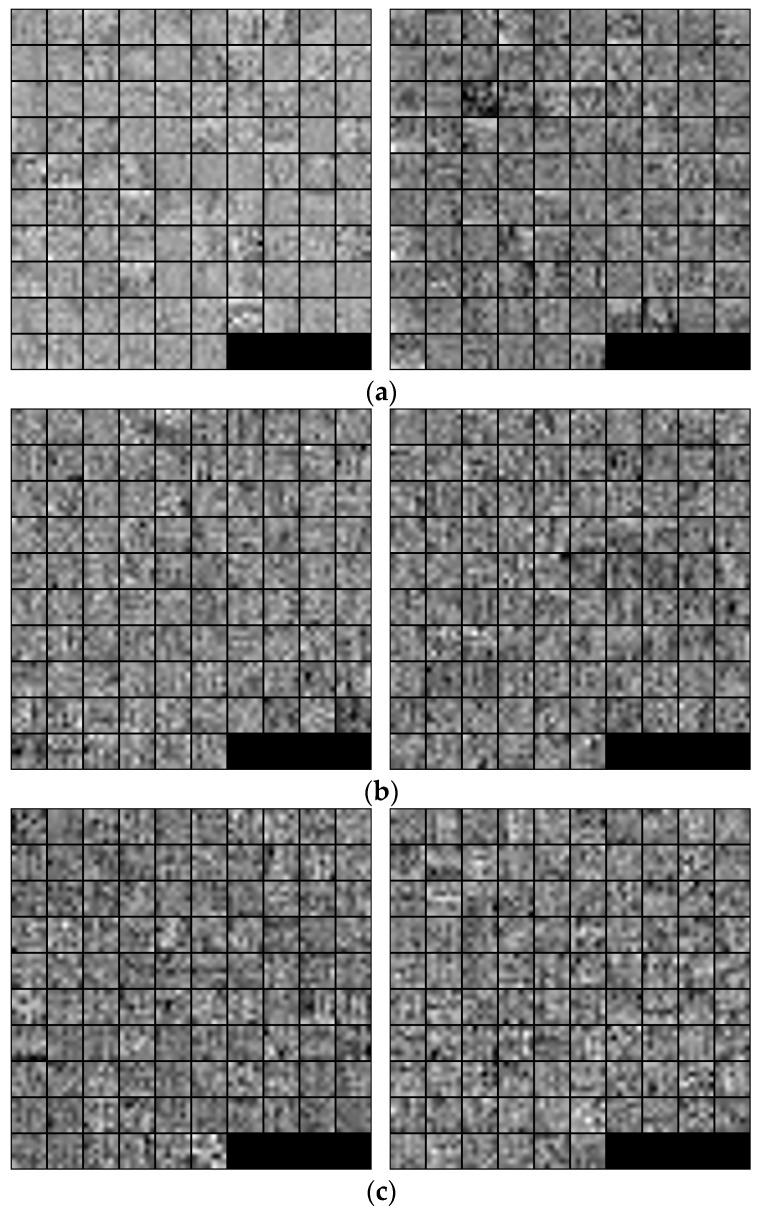
Visualization of filter parameters in the first convolutional layers of the CNN model in each national currency dataset, in which the left and right images are obtained from the trained models on the first and second subsets for two-fold cross-validation, respectively: (**a**) KRW; (**b**) INR; and (**c**) USD.

In Figure 8, we show the 96 trained filters in the first convolutional layers of the trained CNN models for each national currency dataset using two-fold cross-validation. For visualization, the original 7 × 7 × 1 pixel filters were resized by a factor of 5 and the weight values were scaled to the range of unsigned integer number from 0 to 255, corresponding to the gray-scale image intensity values.

With the trained CNN models, we conducted the testing experiments on the datasets of each national currency, in a combination of all the denominations and input directions of the banknote images. The experimental results of the two-fold cross-validation using CNN for each dataset are shown in Table 4, Table 5 and Table 6, and expressed as the confusion matrices between the desired and predicted outputs, namely the actual fitness levels of the banknotes and the fitness-classification results using the trained CNN models. From the testing results on two subsets, we calculated the average accuracy based on the number of accurately classified cases of each subset as the following formula [32]:

(10) $$Avr\_Acc=\frac{GA_{1}+GA_{2}}{N}$$

with *Avr_Acc* the average testing accuracy of the total *N* samples in the dataset, and *GA*_1_ and *GA*_2_ are the number of accurately classified samples (genuine acceptance cases) from the 1st and 2nd fold cross validations, respectively.

Table 4, Table 5 and Table 6 show that the proposed CNN-based method yields good performance with the average testing accuracy of the two-fold cross-validation of approximately 97% in the cases of the KRW and USD, and more than 99% in the case of the INR, even with the merged denominations and input directions of banknote images in each dataset.

**Figure 9:**
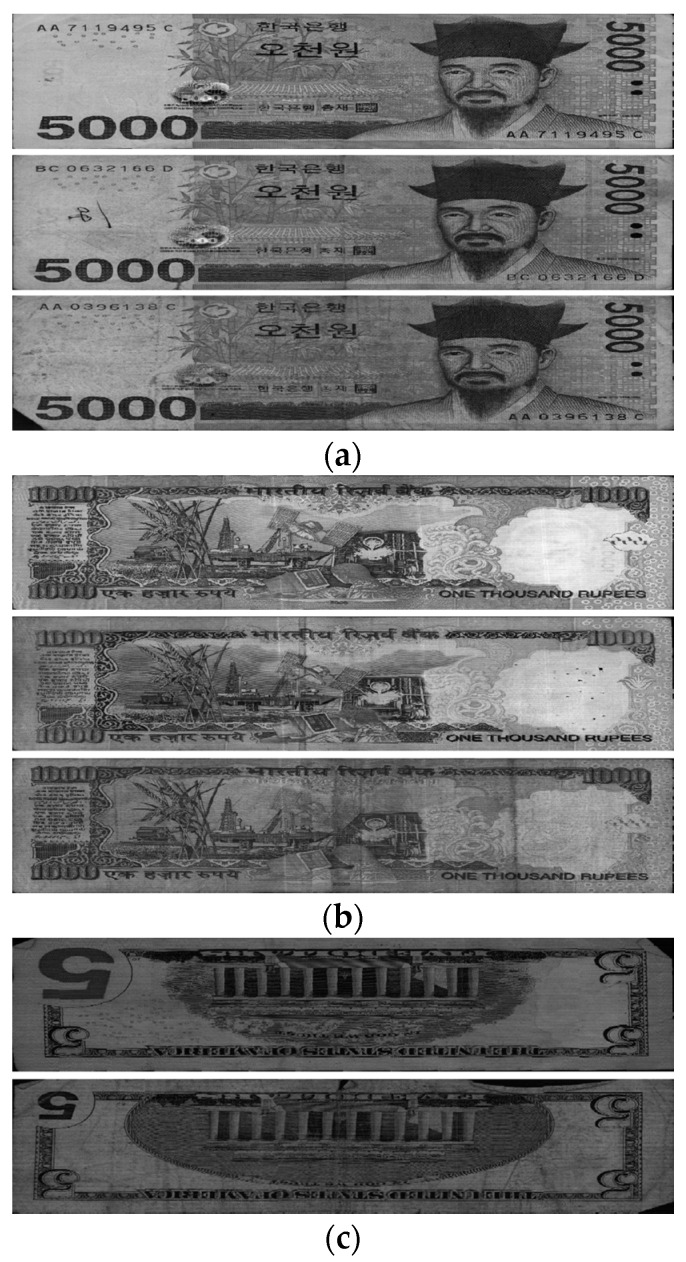
Examples of correctly classified cases by our method of the (**a**) KRW; (**b**) INR; and (**c**) USD datasets. In (**a**,**b**), upper, middle and lower figures show the cases that are the correctly classified fit, normal, and unfit banknotes, respectively. In (**c**), the upper and lower figures are the correctly recognized fit and unfit banknotes, respectively.

In Figure 9, we show the examples of correctly classified cases in the testing results using our proposed method on the KRW, INR, and USD datasets. Figure 9 shows that the degradation degrees in the INR banknotes are clearer to be distinguished among fitness classes of fit, normal, and unfit than that in the case of the KRW. Furthermore, the visible-light banknote images captured in the case of the USD have slightly lower brightness than those of the KRW and INR. This resulted in the highest average classification accuracy in the testing results using our proposed method on the INR dataset compared to that of the KRW and USD.

**Figure 10:**
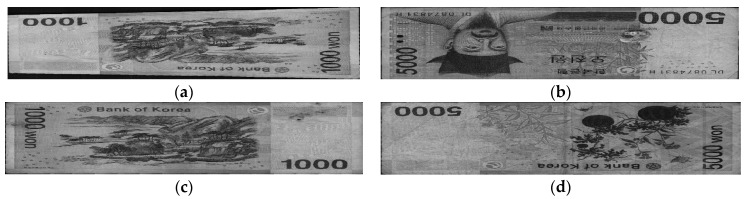
Examples of false recognition cases by our method in the KRW dataset: (**a**) fit banknote misclassified to normal; (**b**) normal banknote misclassified to fit; (**c**) unfit banknote falsely recognized as normal banknote; and (**d**) normal banknote falsely recognized as unfit banknote.

**Figure 11:**
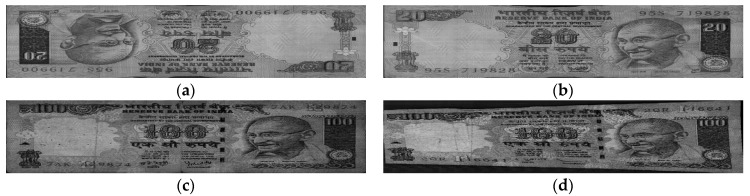
Examples of false recognition cases by our method in the INR dataset: (**a**) fit banknote misclassified to normal; (**b**) normal banknote misclassified to fit; (**c**) unfit banknote falsely recognized as normal banknote; and (**d**) normal banknote falsely recognized as unfit banknote.

**Figure 12:**
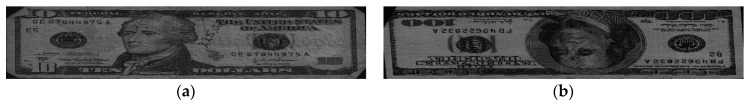
Examples of false recognition cases by our method in the USD dataset: (**a**) fit banknote misclassified to unfit; (**b**) unfit banknote misclassified to fit.

Examples of error cases are also given in Figure 10, Figure 11 and Figure 12 for each of the national currency datasets. As shown in these figures, there were some cases where the input banknotes were incorrectly segmented from the background, as shown in Figure 10a and Figure 11d. This resulted in the banknotes being classified as the classes of lower fitness level. Figure 10c and Figure 11c show that the stained and soiled areas occurred sparsely on the banknotes and occasionally could not be recognized by using only visible-light images as in our method. Banknote images in Figure 11a,b are from the fit and normal classes, respectively; however, besides the similar brightness, both of the banknotes were slightly folded on the upper parts, which affected the classification results. The fit USD banknote in Figure 12a has hand-written marks, whereas the degradation on the unfit banknote in Figure 12b is the fading of texture in the middle of the banknote rather than staining or soiling. These reasons caused the misclassification of fitness level in these cases. In addition, the average classification accuracy of the normal banknotes was the least among the three fitness levels in the case of INR and KRW. This is because of the fact that, the normal banknotes have the middle quality levels, which consist of stained or partly damaged more than fit banknotes but not enough to be replaces by the new ones as the cases of unfit banknotes. This resulted in the largest confusions occurring between normal class and either the fit or unfit classes, and the average classification accuracies in the cases of normal classes in both INR and KRW datasets were the least. 

In the subsequent experiments, we compared the performance of the proposed method with that of the previous studies reported in [7,11]. As both of the previous methods required training, we also performed the two-fold cross-validation in the comparative experiments. Referring to [7], we extracted the features from the gray-level histogram of the banknote image and used the multilayered perceptron (MLP) network as the classifiers, with 95 network nodes in the input and hidden layers. In the case of the comparative experiments using the method in [11], we selected the areas that contain less texture on the banknote images as ROIs, and calculated the means and standard deviation values of the ROIs’ Daubechies wavelet decomposition. Because the fitness classifiers in [11] are the SVM, in the case of the KRW and INR datasets that have three fitness levels, we trained the SVM models using the one-against-all strategy [33]. The experiments with previous methods were implemented using MATLAB toolboxes [34,35].

A comparison of the experimental results between our proposed method and those in previous studies are shown in Table 7, Table 8 and Table 9, in which the fitness-classification accuracies are calculated separately according to denominations and input directions of the banknote images in each national currency. This is because in the previous studies, the fitness-classification models were trained with these manually separated type banknote images. Therefore, although our proposed method does not require the pre-classification of denominations and input directions of the banknote images, we showed the accuracies separately according to these categories for comparison.

Table 7, Table 8 and Table 9 show that the proposed CNN-based fitness classification method outperformed the previous methods in terms of higher average classification accuracy for all the national currency datasets. This can be explained by the disadvantages of each method: the histogram-based method used only the overall brightness characteristic of the banknote images for the classification of fitness levels. This feature was strongly affected by the capturing condition of the sensors. Moreover, degradation might occur sparsely on the banknote, therefore it cannot be easily recognized by the brightness histogram only. The ROI-based method in [11] relied only on the less textured areas on the banknote images. Consequently, if the degradation or damage of the banknote occurs on other areas, it will not be as effective as the proposed method. The CNN-based method has the advantage of the ability to train not only the classifier in the fully connected layer parts but also the filter weights in the convolutional layers, which can be considered as the feature extraction part. As a result, both the feature extraction and classification stages were intensively trained by the training datasets. Moreover, when the whole banknote image is inputted to the CNN architecture, we can make use of all of the available optical characteristics of the banknote for feature extraction. Consequently, owning to the advantages in the feature extraction procedure, the proposed fitness-classification method gave better performance compared to previous methods in terms of higher average accuracy using two-fold cross-validation.

## 5. Conclusions

This study proposed a fitness-classification method using visible-light banknote images and CNN. The fitness level of the banknotes is assigned to three levels for the cases of the KRW and INR, and two levels for the USD banknotes. Our proposed method is designed to classify fitness level regardless of the denominations and input directions of the banknote images. The experimental results on the three datasets of the KRW, INR, and USD banknote images with merged denominations and input directions gave good performances, and showed that the proposed method outperformed the methods in the previous studies, in terms of higher average accuracy with two-fold cross-validation. For future work, we plan to test the proposed method with banknotes from other countries. We also intend to further study the multinational fitness-classification method, which is able to simultaneously recognize the fitness level of banknotes from multiple countries.

## Figures and Tables

**Table 1:** Comparison of the proposed method and previous works on the fitness classification of banknotes.

Category	Method	Advantage	Disadvantage
Using certain regions on banknote image	Using features extracted from various color channels of overlapping regions on banknote images [3,10]. Using DWT for feature extraction from ROIs on visible-light images of banknotes and classifying fitness by SVM [11]. Using fuzzy system for fitness determination based on ROIs on VR and NIRT images of banknotes [12].	Less resource requirement owing to the small sizes of processing areas and features.	Defects and damages can occur on the non-selected regions of the banknote.
Using the whole banknote image	Using the gray-scale histogram of banknote images and classify fitnessusing DTW and SVM [6] or using an NN [7]. Using multiresolutional features of visible and IR images of banknote for recognition [8]. Soiling evaluation based on using image morphological operations and Otsu’s thresholding on banknote images [9].	Make use of all the available characteristics of banknote images for fitness classification.	- Possible data redundancy at the input stage. - Histogram-based methods are affected by imaging conditions and variations in banknote patterns - Pre-classification of banknote’s denomination and input direction is required.
	Fitness classification using a CNN (Proposed method)	Pre-classification of banknote’s denomination and input direction is not required.	Intensive training of the CNN is required.

**Table 2:** Structure of CNN used in our proposed method (unit: pixel).

Layer Type		Size of Kernel	Number of Stride	Padding	Number of Filters	Size of Feature Map
Image Input Layer						115 × 51 × 1
C1	Convolutional Layer	7 × 7 × 1	2	0	96	55 × 23 × 96
	ReLU Layer					
	CCN Layer					
	Max Pooling Layer	3 × 3 × 96	2	0	1	27 × 11 × 96
C2	Convolutional Layer	5 × 5 × 96	1	2	128	27 × 11 × 128
	ReLU Layer					
	CCN Layer					
	Max Pooling Layer	3 × 3 × 128	2	0	1	13 × 5 × 128
C3	Convolutional Layer	3 × 3 × 128	1	1	256	13 × 5 × 256
	ReLU Layer					
C4	Convolutional Layer	3 × 3 × 256	1	1	256	13 × 5 × 256
	ReLU Layer					
C5	Convolutional Layer	3 × 3 × 256	1	1	128	13 × 5 × 128
	ReLU Layer					
	Max Pooling Layer	3 × 3 × 128	2	0	1	6 × 2 × 128
F1	Fully Connected Layer					4096
	ReLU Layer					
F2	Fully Connected Layer					2048
	ReLU Layer					
	Dropout Layer					
F3	Fully Connected Layer					2 or 3 (Number of Fitness Levels)
	Softmax Layer					

**Table 3:** Number of banknote images in each national currency database.

Fitness Levels		KRW	INR	USD
Fit	Number of Images	10,084	11,909	2907
	Number of Images after Data Augmentation	30,252	71,454	61,047
Normal	Number of Images	12,430	7952	N/A
	Number of Images after Data Augmentation	37,290	47,712	N/A
Unfit	Number of Images	11,274	2203	642
	Number of Images after Data Augmentation	33,822	13,218	45,582

**Table 4:** Confusion matrices of testing results on the KRW banknote fitness dataset using the proposed method. The 1st Testing Results and 2nd Testing Results mean the results of the testing on the 1st and 2nd subsets of banknote images in the two-fold cross-validation method, respectively (unit: %).

**1st Testing Results**		**Predicted Results**		
		Fit	Normal	Unfit
**Desired Outputs**	Fit	98.830	1.170	0.000
	Normal	3.460	93.610	2.929
	Unfit	0.035	2.148	97.817
**2nd Testing Results**		**Predicted Results**		
		Fit	Normal	Unfit
**Desired Outputs**	Fit	96.827	3.173	0.000
	Normal	0.579	98.890	0.531
	Unfit	0.000	2.677	97.323
Average Accuracy		97.612		

**Table 5:** Confusion matrices of the testing results on the INR banknote fitness dataset using the proposed method. The 1st Testing Results and 2nd Testing Results mean the same as those in Table 4 (unit: %).

**1st Testing Results**		**Predicted Results**		
		Fit	Normal	Unfit
**Desired Outputs**	Fit	99.832	0.168	0.000
	Normal	0.705	99.094	0.201
	Unfit	0.000	0.548	99.452
**2nd Testing Results**		**Predicted Results**		
		Fit	Normal	Unfit
**Desired Outputs**	Fit	99.882	0.118	0.000
	Normal	0.377	99.472	0.151
	Unfit	0.000	0.000	100.000
Average Accuracy		99.637		

**Table 6:** Confusion matrices of the testing results on the USD banknote fitness dataset using the proposed method. The 1st Testing Results and 2nd Testing Results mean the same as those in Table 4 (unit: %).

**1st Testing Results**		**Predicted Results**	
		Fit	Unfit
**Desired Outputs**	Fit	99.724	0.276
	Unfit	15.142	84.858
**2nd Testing Results**		**Predicted Results**	
		Fit	Unfit
**Desired Outputs**	Fit	99.520	0.480
	Unfit	14.769	85.231
Average Accuracy		96.985	

**Table 7:** Comparison of fitness-classification accuracy by our proposed method with that of previous studies on the KRW banknote dataset. Denom. and Dir. are denominations and directions, respectively. The 1st Testing Results and 2nd Testing Results mean the same as those in Table 4 (unit: %).

Denom.	Dir.	Method Based on Gray-level Histogram and MLP [7]			Method Based on DWT and SVM [11]			Proposed Method		
		1st Testing Accuracy	2nd Testing Accuracy	Average Accuracy	1st Testing Accuracy	2nd Testing Accuracy	Average Accuracy	1st Testing Accuracy	2nd Testing Accuracy	Average Accuracy
KRW 1000	A	55.974	63.459	59.719	68.926	72.682	70.805	94.930	94.536	94.733
	B	86.504	68.650	77.577	80.037	78.116	79.077	95.408	96.954	96.181
	C	76.631	62.961	69.793	45.625	50.733	48.180	97.521	98.282	97.902
	D	78.058	81.823	79.942	56.796	60.640	58.719	96.893	96.946	96.920
KRW 5000	A	84.766	96.859	90.814	85.203	85.035	85.119	96.552	99.476	98.014
	B	79.472	93.528	86.502	82.734	84.851	83.793	95.683	97.795	96.739
	C	78.459	99.072	88.765	70.427	69.777	70.102	98.514	99.536	99.025
	D	89.157	86.254	87.705	76.857	80.883	78.871	96.993	98.134	97.564
Average Accuracy		80.487			72.230			97.162		

**Table 8:** Comparison of fitness-classification accuracy by our proposed method with that of previous studies on the INR banknote dataset. Denom., Dir., 1st Testing Results and 2nd Testing Results mean the same as those in Table 7 (unit: %).

Denom.	Dir.	Method Based on Gray-level Histogram and MLP [7]			Method Based on DWT and SVM [11]			Proposed Method		
		1st Testing Accuracy	2nd Testing Accuracy	Average Accuracy	1st Testing Accuracy	2nd Testing Accuracy	Average Accuracy	1st Testing Accuracy	2nd Testing Accuracy	Average Accuracy
INR 10	A	100.000	100.000	100.000	89.981	91.715	90.848	100.000	100.000	100.000
	B	100.000	100.000	100.000	90.559	91.329	90.944	100.000	100.000	100.000
	C	100.000	100.000	100.000	96.935	97.323	97.129	100.000	100.000	100.000
	D	100.000	100.000	100.000	97.359	99.245	98.302	100.000	100.000	100.000
INR 20	A	92.437	93.855	93.147	84.594	86.592	85.594	100.000	99.441	99.720
	B	91.292	93.017	92.157	85.955	87.430	86.695	98.876	99.441	99.160
	C	93.277	91.922	92.598	93.277	93.315	93.296	99.720	99.721	99.721
	D	92.877	95.184	94.034	92.308	92.068	92.188	99.715	100.000	99.858
INR 50	A	99.346	99.674	99.511	93.464	92.508	92.985	100.000	100.000	100.000
	B	99.674	100.000	99.837	90.228	88.312	89.268	100.000	100.000	100.000
	C	100.000	100.000	100.000	93.069	93.443	93.257	100.000	100.000	100.000
	D	99.676	100.000	99.839	90.939	93.248	92.097	100.000	100.000	100.000
INR 100	A	99.140	98.650	98.895	91.646	89.816	90.731	99.017	99.509	99.263
	B	99.026	98.660	98.843	90.012	89.769	89.890	99.513	99.756	99.635
	C	97.340	97.582	97.461	89.480	90.085	89.782	99.637	100.000	99.819
	D	98.315	98.798	98.557	91.697	90.745	91.221	99.519	99.639	99.579
INR 500	A	88.153	88.353	88.253	86.747	87.952	87.349	99.398	99.598	99.498
	B	89.421	88.845	89.133	86.028	86.255	86.142	98.403	99.602	99.003
	C	90.041	89.697	89.868	88.211	87.879	88.045	97.967	98.990	98.480
	D	85.859	88.531	87.198	88.081	87.726	87.903	99.394	99.396	99.395
INR 1000	A	97.166	95.547	96.356	76.923	76.923	76.923	99.190	99.595	99.393
	B	97.590	96.825	97.206	78.715	79.365	79.042	100.000	100.000	100.000
	C	96.825	96.047	96.436	88.889	89.723	89.307	100.000	99.605	99.802
	D	97.266	98.438	97.852	85.938	85.938	85.938	99.609	100.000	99.805
Average Accuracy		96.274			89.952			99.637		

**Table 9:** Comparison of fitness-classification accuracy by our proposed method with that of previous studies on the USD banknote dataset. Denom., Dir., 1st Testing Results and 2nd Testing Results mean the same as those in Table 7 (unit: %).

Denom.	Dir.	Method Based on Gray-level Histogram and MLP [7]			Method Based on DWT and SVM [11]			Proposed Method		
		1st Testing Accuracy	2nd Testing Accuracy	Average Accuracy	1st Testing Accuracy	2nd Testing Accuracy	Average Accuracy	1st Testing Accuracy	2nd Testing Accuracy	Average Accuracy
USD 5	A	96.774	82.540	89.600	75.807	76.191	76.000	98.387	96.825	97.600
	B	78.723	82.979	80.851	74.468	76.596	75.532	87.234	95.745	91.489
	C	81.395	75.000	78.161	44.186	56.818	50.575	95.349	93.182	94.253
	D	95.652	89.130	92.391	71.739	76.087	73.913	91.304	100.000	95.652
USD 10	A	80.682	82.022	81.356	88.636	88.764	88.701	96.591	92.135	94.350
	B	80.851	92.632	86.772	94.681	94.737	94.709	100.000	100.000	100.000
	C	73.973	68.919	71.429	65.753	56.757	61.224	93.151	95.946	94.558
	D	93.590	100.000	96.835	89.744	83.750	86.709	94.872	100.000	97.468
USD 50	A	91.358	96.341	93.865	82.716	83.537	83.129	95.062	98.780	96.933
	B	99.394	98.795	99.094	93.939	90.964	92.447	96.364	96.988	96.677
	C	91.837	92.568	92.203	93.197	92.568	92.881	97.959	96.622	97.288
	D	91.156	91.892	91.525	89.796	89.189	89.492	95.918	93.919	94.915
USD 100	A	98.137	96.914	97.523	86.335	86.420	86.378	100.000	98.765	99.381
	B	95.513	94.267	94.888	87.820	87.898	87.859	98.718	94.904	96.805
	C	92.157	94.771	93.464	90.196	90.196	90.196	99.346	96.732	98.039
	D	94.483	87.671	91.065	91.034	90.411	90.722	99.310	98.630	98.969
Average Accuracy		91.462			85.940			96.985		
